# Purification of Sesquiterpenes from *Saussurea Lappa* Roots by High Speed Counter Current Chromatography

**DOI:** 10.22037/ijpr.2019.1100755

**Published:** 2019

**Authors:** Kun Cao, Wei Qian, Yi Xu, Zhen Zhou

**Affiliations:** a *Pharmaceutical and Biological Engineering College, Chongqing University of Technology, Chongqing 400054, P.R. China*.; b *College of Chemistry and Chemical Engineering, Chongqing University, Chongqing 400030, P.R. China. *; c *Defense Key Disciplines Lab of Novel Micro-nano Devices and System Techonlogy, Chongqing, 400030, P.R. China.*; d *International Research and Development Center of Micro-nano Systems and New Materials Technology, 400030, P.R. China.*

**Keywords:** Saussurea lappa Roots, Sesquiterpenes lactones, Select solvent system, HSCCC, Orthogonal test

## Abstract

Sesquiterpenes lactones including costunolide and dehydrocostus lactone, were isolated from *Saussurea lappa *roots, which had exhibited a wide range of biological activities such as anti-cancer, anti-inflammatory, antiulcer, and immunomodulatory activities. High-speed counter-current chromatography (HSCCC) was applied for the rapid preparative isolation of sesquiterpenes lactones from *Saussurea lappa* roots. A solvent optimization method for HSCCC was presented, i.e. the separation factors of compounds after the partition coefficient (K) of solvent system should be investigated. Using this method, 150 mg of costunolide, 140 mg of dehydrocostus and 15 mg of 10-methoxy-artemisinic acid with purities of 95%, 98%, and 98% were obtained within 150 min. The structures of three compounds were identified by mass spectrum (MS) and nuclear magnetic resonance (NMR) spectroscopy. These results offered an efficient strategy for separation of potentially health-relevant phytochemicals from *Saussurea lappa* roots.

## Introduction

Muxiang is the roots of *Saussurea lappa* C.B. Clarke, which has been widely used in India, China, and Japan for the treatment of asthma, cough, diarrhea, vomit, indigestion, colic, cholecystitis, cancer, and hepatitis (1-2). Sesquiterpenes lactones, such as costunolide and dehydrocostus lactone, were representative active compounds of *Saussurea lappa (S. lappa)* roots (3). It displayed multiple biological activities such as anticancer, anti-inflammatory, antiulcer, hepatoprotective, and immunomodulatory activities (4). Moreover, they are the major index compounds for the quality control of *S. lappa* roots.

 High-speed counter-current chromatography (HSCCC) is a unique form of free liquid-liquid partition chromatography, which can eliminate irreversible adsorption of sample on a stationary phase in conventional column chromatography (4-6). HSCCC has been widely used in the separation and purification of natural products with high sample recovery and loading capacity, low solvent consumption, and acceptable efficiency (7-9). HSCCC solvent systems of these separations were determined only according to the partition coefficient (K) which was analyzed by HPLC. However, no report has been published on the factors of the solvent system, revolution speed, flow rate, and other factors in depth during the separation, which was the guidance for solvent system.

In the present study, an efficient HSCCC method was developed for fast isolation of sesquiterpenoid lactones from *S. lappa* roots. A solvent system optimization method of HSCCC was established, combined partition coefficient (K) determination with HSCCC analysis. Based on this approach, a ternary solvent system was selected and applied for the HSCCC and two index compounds costunolide and dehydrocostus were isolated from *S. lappa* roots, along with another sesquiterpenoid. Their chemical structures were elucidated by NMR, High Resolution Mass Spectrometer (HRMS) experiments. The results were discussed herein.

## Experimental

*Apparatus*

Separation was performed by the Opti-Chrome™-A double-action column semi-preparation HSCCC instrument (Counter Current Technology Co., Ltd., Jiangsu, China); The instrument was a fully automated system consisting of a 2 polytetrafluroethylene (PTFE) preparative coils (i.d. of tube, 1.59 mm; revolution radius, 80 cm; range of β, 0.50-0.80; total volume, 360 mL) and a 20-mL sample loop. The HSCCC system was equipped with a model UV-3000 detector. Separating effect could be predicted by a FastChrome-30 analytical HSCCC instrument (Counter-Current Technology Co., Ltd., Jiangsu, China) with a PTFE preparative coils (total volume, 25 mL; range of β, 0.56-0.91), a 1 mL sample loop; NMR spectra were obtained on an Agilent 600 MHz DD2 NMR spectrometer (Agilent Technologies Inc., USA); The partition coefficients were measured on an Agilent 1260 HPLC system (Agilent Technologies Inc., USA).

*Plant material and Reagents*

All chemicals including organic solvents which were for sample preparation and HSCCC were of analytical grade and purchased from Chuandong Chemical Factory (Chongqing, China). Methanol and acetonitrile which were for HPLC analysis was of chromatographic grade (Adamas-beta Chemical Reagent Co., Ltd. Switzerland) and water was distilled. 

*Saussurea lappa *roots were obtained from a local drug store (Chongqing, China) and authenticated by Pro. Jingou Ji, department of pharmacy, Chongqing University.

*Preparation of crude extract*

Two kilograms dried roots of *S. lappa* were cut into chunks and refluxing extracted with 5 L water for 3 h (3×), the combined aqueous extract was evaporated under reduced pressure. The extracts were subsequently dissolved in water, partitioned with equal volume petroleum ether (PE) (60-90 °C, 3×). The PE solutions were evaporated to generate 25.3 g.

*Evaluation of K value*

According to Xueli Cao’s research, the partition coefficient should be between 0.2-5 (10). The K values of two compounds were determined by HPLC as follows: 5 mg crude extract was added into a series of pre-equilibrated two-phase solvent (10 mL), and the solution was then fully shaken to reach the partition equilibrium. Subsequently, the upper and the lower phases were analyzed by HPLC. The K value was expressed as the peak area of the target compound in the upper phase divided by that in the lower phase. 

*Verification of solvent systems by analytical HSCCC*

Seven solvent systems were primarily determined, including petroleum ether-methanol-water (5:6:4), petroleum ether-methanol-water (5:6.5:3.5), petroleum ether-ethyl acetate-methanol-water (5:1:6.5:3.5), petroleum ether-ethyl acetate-methanol-water (5:5:6.5:3.5), petroleum ether-acetone-methanol-water (5:1:6.5:3.5), petroleum ether-methanol-water (5:7:3) and petroleum ether-methanol-water (5:7.5:2.5). These solvent systems were prepared respectively by mixing the solvents in a separation funnel according to the volume ratios and thoroughly equilibrated by shaking them vigorously. Then, they were left overnight and two phases were separated and degassed by sonication for 30 min prior to use. The sample solution for analysis was prepared as follows: 80 mg crude extract was dissolved in 10 mL solvent mixture of upper and lower phase (1:1, v/v) of the two-solvent system.

Analytical HSCCC separation was performed as described previously (11). The detection wavelength in this study was 254 nm. The optimal solvent system was determined by comparing resolution and equilibration time of two-phase solvent system.

*Optimization separation and purification conditions of HSCCC*

The solvent systems, revolution speed and flow rate had obvious influence on the resolution, separating time and retention rate in actual separation. And peak width and retention rate of the stationary phase were two important factor, which were directly related to theoretical plate number, resolution, separation time, separation efficiency, the purity of product, sample weight and the yield of separation product. 

For the preparative HSCCC separation, a L9 (3^4^) orthogonal test was applied to optimize experimental conditions which included the solvent system (A), revolution speed (B), and flow rate (C). Based on the single factor experiments, revolution speed was selected from 700 to 1100 rpm and flow rate was selected from 1 to 5 mL min^-1^. Column temperature was selected at room temperature. The levels of these three factors were shown in Table 1. These solvent systems were shaken vigorously in a separatory funnel and equilibrated at room temperature for overnight, respectively. After each layer was degassed by sonication for 30 min, the lower phase was used as the mobile phase, and the upper phase was used as the stationary phase.

The preparative HSCCC separation protocol was described previously (11). The injection amount and detection wavelength in this study were 500 mg and 254 nm, respectively. The fractions were collected according to the elution profile and evaporated with a rotary evaporator. The sample residues were stored at –20 °C before HPLC and ^1^H and ^13^C NMR analyses. Peak resolution was computed according to the chromatogram.

*Analysis of preparation product*

HPLC analyses of the crude sample and the HSCCC peak fractions were performed with a Kromasil C_18_ column (250 × 4.6 mm, 5 μm) at 25 °C. Methanol- water (85: 15, v/v) was used as the mobile phase. The flow rate was 0.8 mL min^-1^ and the column effluent was monitored by use of the DAD at 254 nm, and the injection volume of sample was 10 μL. Identification of each isolated target compound was carried out by ^1^H-NMR, ^13^C-NMR, ^1^H-^1^H Correlation Spectroscopy (COSY), Heteronuclear Single Quantum Correlation Spectroscopy (HSQC), Heteronuclear Multiple Quantum Correlation Spectroscopy (HMQC).

## Results and Discussion

HSCCC is a unique efficient liquid-liquid partition chromatography separation technique, which eliminates irreversible adsorption of samples on solid support in the conventional separation methods. In HSCCC, the separation of compounds is based on the partition coefficient (K) in two phases. Suitable retention rate and partition coefficient are important factors for a satisfied separation process. So, it is important to investigate the factors, such as solvent systems, revolution speed, and flow rate and so on, which have a great impact on retention rate and partition coefficiency. 

*Measurement of Partition coefficient (K)*

The partition coefficients (K) of compounds in two-phase solutions and the retention on stationary phase were the two most important factors for stable and reliable solvent systems of HSCCC. According to the peak area of dehydrocostus lactone and costunolide measured by HPLC in two-phase solvent systems, K values of seven solvent systems were expressed as the peak area of the target compound in the upper phase divided by that in the lower phase, and the results were shown in Table 2.

**Table 1:** Factor-levels for the orthogonal experiments

**Factor-levels**	**A**	**B (rpm)**	**C (mL min** **-1** **)**
1	Petroleum ether-methanol-water (5:7:3, v/v/v)	800	2
2	Petroleum ether–ethyl acetate–methanol–water (5:1:6.5:3.5, v/v/v/v)	900	2.5
3	Petroleum ether-methanol-water (5:6.5:3.5, v/v/v)	1000	3

**Table 2:** K values of dehydrocostus lactone and costunolide in different solvent systems

**Solvent system (v/v)**	**K** **2 ** **(costunolide)**	**K** **3 ** **(dehydrocostus lactone)**	**α (K** **3** **/K** **2** **)**
petroleum ether-methanol-water (5:6:4)	3.05	5.91	1.94
petroleum ether-methanol-water (5:6.5:3.5)	2.21	4.06	1.84
petroleum ether-ethyl acetate-methanol-water (5:1:6.5:3.5)	1.70	3.19	1.88
petroleum ether-ethyl acetate-methanol-water (5:5:6.5:3.5)	1.95	2.46	1.26
Petroleum ether-acetone-methanol-water (5:1:6.5:3.5)	1.30	2.44	1.87
petroleum ether-methanol-water (5:7:3)	0.99	1.90	1.92
petroleum ether-methanol-water (5:7.5:2.5)	0.46	0.87	1.89

**Table 3:** Separating results in different conditions by semi-preparation HSCCC

**Test NO.**	**Solvent system**	**Revolution speed (rpm)**	**Flow rate (mL min** ^-1^ **)**	**Retention**	**Total time**	Retention volume				**Peak width**		**R**	**K**_2-3_
						(peak 1)	(peak 2)		(peak 3)	(peak 2)	(peak 3)		
1	A1	B1	C1	80.6%	240min	134.6	263.4	440		29.6	58.7	3.59	1.28
2	A1	B2	C2	81.6%	180min	136	256.25	419.5		22.1	40.3	5.23	1.20
3	A1	B3	C3	81.1%	146min	146.4	256.5	405.3		18.3	33.2	5.78	1.16
4	A2	B1	C2	76.7%	298min	233.75	440.75	679.5		47.0	73.0	3.98	2.16
5	A2	B2	C3	79.4%	250min	207.3	437.7	670.5		42.1	55.5	4.77	2.09
6	A2	B3	C1	91.4%	350min	206.8	440.6	660.5		65.1	85.5	2.92	1.91
7	A3	B1	C3	73.9%	230min	176.4	366.9	642		33.5	56.4	6.12	2.06
8	A3	B2	C1	86.4%	342min	169.6	365.8	639.6		48.2	86.6	4.06	1.90
9	A3	B3	C2	84.7%	480min	201.5	370.8	655.4		45.4	66.8	5.07	1.97

**Table 4:** Results of variance analysis about the peak width of peak **3** and the retention rate

**Factors**	**the peak width of peak 3**				**the retention rate**			
	Sum of squares of deviations	Degree of freedom	F value	Significance	Sum of squares of deviations	Degree of freedom	F value	Significance
A	1414.52	2	88.34	**	2.98	2	1.37	
B	5.43	2	0.32		114.94	2	52.82	**
C	1237.78	2	73.80	**	98.57	2	45.30	**
Error	28.12	2			1.38	2		

**Table 5:** The comparison of the various K-values (average value, n = 5, RSD < 1.2%).

**Solvent system**	**Preparative HSCCC**		**α **_Pre_	**HPLC**		**α **_HPLC_	**Analytical HSCCC**		**α **_Ana_
	**K**_2_	**K**_3_		**K**_2_	**K**_3_		**K**_2_	**K**_3_	
A1	0.65	1.21	1.86	0.99	1.90	1.92	0.73	1.32	1.81
A2	1.27	2.05	1.61	1.70	3.19	1.87	1.10	1.85	1.68
A3	1.03	1.98	1.92	2.21	4.06	1.84	0.93	1.76	1.89

**Figure 1:**
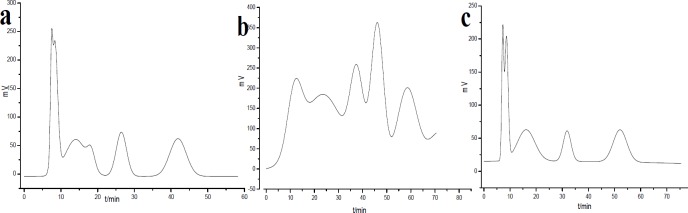
HSCCC chromatogram of PE extract of *Saussurea lappa* roots using different solvent systems. Two-phase solvent systems: (a) petroleum ether-methanol-water (5:7:3); (b) petroleum ether-ethyl acetate-methanol-water (5:1:6.5:3.5); (c) petroleum ether-methanol-water (5:6.5:3.5); revolution speed: 1800 rpm; detection wavelength: 254 nm; flow rate: 1 mL/min; sample injection: 1 mL

**Figure 2:**
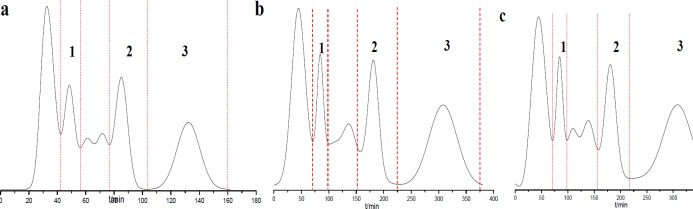
Semi-preparation HSCCC chromatogram of PE extract of *Saussurea lappa *roots using different solvent systems. (a) petroleum ether-methanol-water (5:7:3), 3 mL/min, 1000 rpm; (b) petroleum ether-methanol-water (5:6.5:3.5), 2 mL/min, 900 rpm; (c) petroleum ether-ethyl acetate-methanol-water (5:1:6.5:3.5), 2.5 mL/min, 800 rpm. **1**: 10-methoxy-artemisinic acid, **2**: costunolide, **3**: dehydrocostus lactone; detection wavelength: 254 nm; sample injection: 20 mL

**Figure 3:**
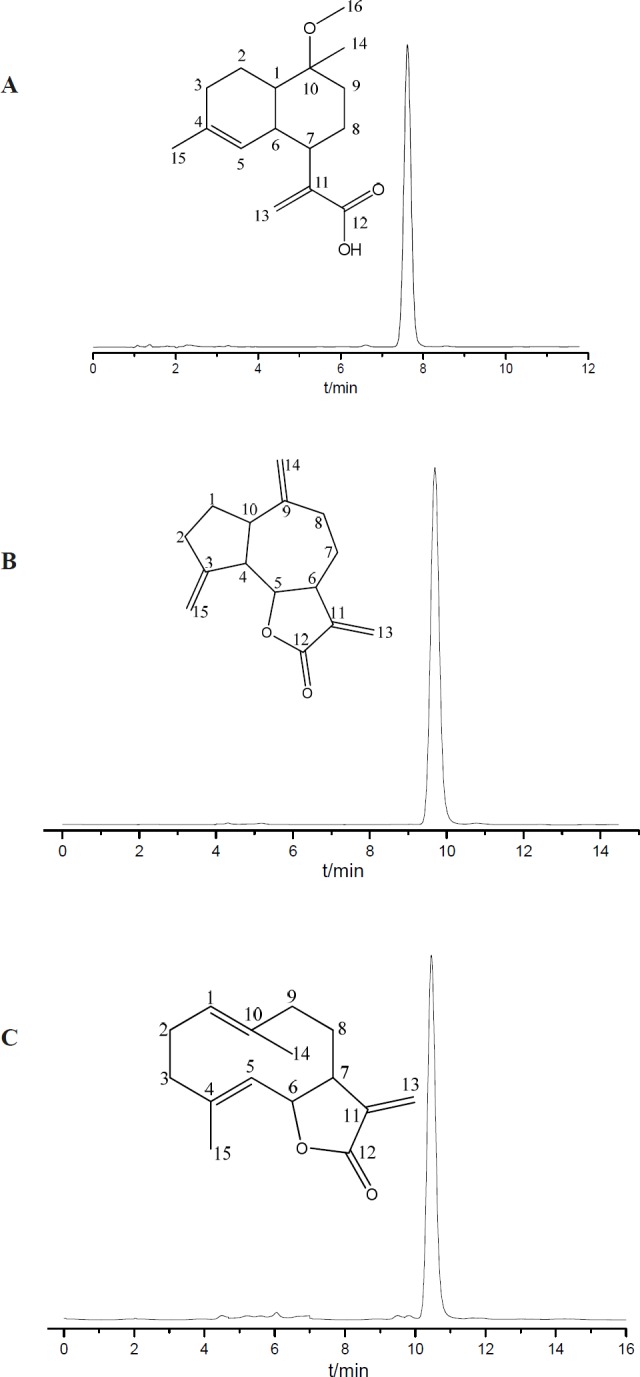
HPLC analyses and structures of the fractions obtained from *Saussurea lappa* roots by HSCCC. (A) 10-methoxy-artemisinic acid; (B) costunolide; (C) dehydrocostus lactone

**Figure 4.**
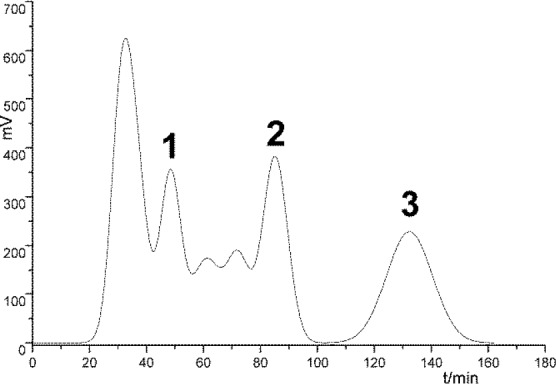
The HSCCC chromatogram of petroleum ether- soluble fraction of *Saussurea lappa *Roots

*Optimization of separation conditions and preparation of target compounds*

To improve the preparation efficiency, PE extract of *S. lappa* roots was analyzed by HSCCC according to the results in Table 2. As a result, petroleum ether-methanol-water (5:7:3), petroleum ether-ethyl acetate-methanol-water (5:1:6.5:3.5) and petroleum ether-methanol-water (5:6.5:3.5) were selected for the orthogonal experiments by preparative HSCCC, taking account some factors as separating time, good peak shape, high resolution, and stable baseline (Figure 1).

During the optimization of HSCCC conditions, an orthogonal experiment was applied to investigate the separation factors solvent system (A), revolution speed (B), and flow rate (C) of target compounds after the K values of the solvent systems. Based on the single factor experiments, the revolution speed was selected from 700 to 1100 rpm and the flow rate was selected from 1 to 5 mL min^-1^. Column temperature was selected at room temperature.

Through comprehensive analysis and discussion (Tables 3 and 4; Figure 2), the best separation condition was selected as A_1_B_3_C_3_ (Figure 2), in which the solvent system was petroleum ether-methanol-water (5:7:3), and retention rate was 1000 rpm and flow rate was 3 mL min^-1^. Under the optimal conditions, the separation was performed within 150 min and the prepared compounds with high purities and high yields were obtained.

The purity of compounds **1**, **2,** and **3 **was 98%, 98%, and 95%, respectively, as determined by HPLC (Figure 3). The amount of the three separated products in one-step preparation process could reach 10-20 mg, 140-150 mg, and 150-180 mg, respectively. Compared with references 4 and 5, the proposed HSCCC strategy can realize the separation more rapidly and efficiently.

*Discussion about determination of partition coefficient (K)*

The partition coefficient (K) was calculated according to the polarity of each solvent. The calculated equation was shown in Equation 1:

K= (V_R_-V_sf_)/V_s _(12) (1)

Where V_R_ was the retention volume of products; V_sf_ was the volume of mobile phase in the column; V_s_ was the volume of stationary phase in the column. Based on the formula, the K values of dehydrocostus lactone and costunolide were calculated according to Equation 1, and shown in Table 5.

Obviously, there was a significant difference between real and measured partition coefficient, and the measured K value was greater than the real value, but separation factors of them (α = K_2_/K_1_) were similar. It showed that the capacity of solutes dissolved in mobile phase in the operation situation of HSCCC was better than in the static situation (13-14). This K value can’t describe the real situation that solutes were distributed in two-phase solvent system, and it only can display a certain trend. This means that the retention volume of products can’t be predicted by this K value. However, separation factor which was measured by HPLC will be helpful to predict the resolution of products (15). Therefore, the separation factors of target compounds should be investigated at first, then the solvent systems which K value was in the range of 0.2-5 were applied as the solvent systems of analytical HSCCC to verify the real separation efficiency. 

The K value measured by analytical HSCCC is closer to the K value measured by preparative HSCCC. Therefore, the analytical HSCCC experiments visually displayed the separating process, and it can help to predict separation efficiency by preparative HSCCC more accurately.

*Structure elucidation of the three compounds*

Under the optimal preparative HSCCC conditions, three sesquiterpenoid lactones were isolated and purified from petroleum ether (PE) extract of *S. lappa* roots in 150 minutes (Figure 4). The structures of these compounds were identified as 10α-methoxyartemisinic acid (**1**), costunolide (**2**), and dehydrocostus lactone (**3**). The chemical structure identification of the three compounds was carried out by MS, ^1^H-NMR, ^13^C-NMR spectra as followers (Supplementary file, Figures S1-S9).

Peak **1**. ^1^H NMR (600 MHz, CDCl_3_), δ (ppm): 6.32 (1H, s, H-13), 5.31 (1H, s, H-13), 5.16 (1H, brs, H-5), 3.20 (3H, s, H-16), 2.87 (1H, m, H-7), 2.78 (1H, m, H-6), 1.99 (1H, m, H-2), 1.95 (1H, m, H-1), 1.56 (3H, s, H-15), 1.19 (3H, s, H-14). ^13^C NMR (600 MHz, CDCl_3_), δ (ppm): 172.92 (C-12), 143.73 (C-11), 135.54 (C-4), 125.25 (C-13), 121.73 (C-5), 76.73 (C-10), 40.46 (C-1), 40.20 (C-7), 35.63 (C-6), 34.49 (C-9), 28.91 (C-3), 24.06 (C-8), 21.62 (C-2). Comparing the data with the literature (16), peak **1** was identified as 10α-methoxyartemisinic acid.

Peak **2**.^ 1^H NMR (500 MHz, CDCl_3_), δ (ppm): 6.25 (1H,d, J = 3.5 Hz, H-13a), 5.51 (1H, d, J = 3.0 Hz, H-13b), 4.85 (1H, dd, J = 11.0, 4.0 Hz, H-1), 4.73 (1H, d, J = 10.0 Hz, H-5), 4.57 (1H, t, J = 9.5 Hz, H-6), 1.68 (3H, s, H-15), 1.41 (3H, s, H-14). ^13^C NMR (500 MHz, CDCl_3_), δ (ppm): 170.59 (C-12), 141.60 (C-11), 140.23 (C-4), 137.10 (C-10), 127.42 (C-5), 127.18(C-1), 119.77 (C-13), 82.04 (C-6), 50.55 (C7), 41.12 (C-3), 39.60 (C-9), 28.18 (C-2), 26.33 (C-8), 17.48 (C-15), 16.24 (C-14). Comparing the data with the literature (17), peak **2** was identified as costunolide.

Peak **3**. ^1^H NMR (500 MHz, CDCl_3_), δ (ppm): 6.18 (1H,d, J = 3.5 Hz, H-13a), 5.45 (1H, d, J = 3.0Hz, H-13b), 5.24 (1H, brs, H-15a), 5.03 (1H,brs, H-15b), 4.87 (1H, brs, H-14a), 4.78 (1H, brs, H-14b), 3.95 (1H, t, J = 9.5 Hz, H-5). ^13^C NMR (500 MHz, CDCl_3_), δ (ppm): 170.33 (C-12), 151.39 (C-11), 149.35 (C-3), 139.87 (C-9), 120.26 (C-13), 112.70 (C-15), 109.65 (C-14), 85.35 (C-5), 52.13 (C-4), 47.70 (C-6), 45.22 (C-10), 36.39 (C-2), 32.72 (C-8), 31.05 (C-7), 30.41 (C-1). Comparing the data with the literature (17), peak **3** was identified as dehydrocostus lactone. 

## Conclusion

In this paper, a method for selecting optimal solvent system was proposed and applied to isolate and prepare active compounds by preparative HSCCC. The partition coefficients (K) of target compounds in different solvents were measured by HPLC, and then the real effects in different solvents were verified by analytical HSCCC, finally an orthogonal test was applied to select the optimal solvents, revolution speed, and flow rate. As a result, the solvents of petroleum ether- methanol- water (5:7:3, v/v/v), the revolution speed of 1000 RPM and the flow rate of 3 mL·min^-1^ were selected, 150 mg costunolide, 140 mg dehydrocostus and also, 15 mg 10-methoxy-artemisinic acid were obtained with purities of 95%, 98%, and 98% in 150 min, respectively.

These results offered an efficient strategy for isolation of potentially health-relevant phytochemicals from *S. lappa* roots. This method might be used for further chemical researches and pharmacological studies or as reference substances.
