# Supplementary material for: Purification of Sesquiterpenes from Saussurea Lappa Roots by High Speed Counter Current Chromatography
Source: Iran J Pharm Res. 2019 Summer;18(3):1499–507. doi: 10.22037/ijpr.2019.1100755 (PMC6934979; doi:10.22037/ijpr.2019.1100755)
Supplement: Supplement [file ijpr-18-1499-s001.pdf]

Supplementary Materials for

Purification of Sesquiterpenes from *Saussurea lappa* Roots by High Speed Counter Current Chromatography

Kun Cao, Wei Qian, Yi Xu* and Zhen Zhou

*To whom correspondence should be addressed. E-mail: xuyibbd@sina.com

Volume 18, Issue 3 (Summer 2019)

This PDF file includes:

Figures S1-S10

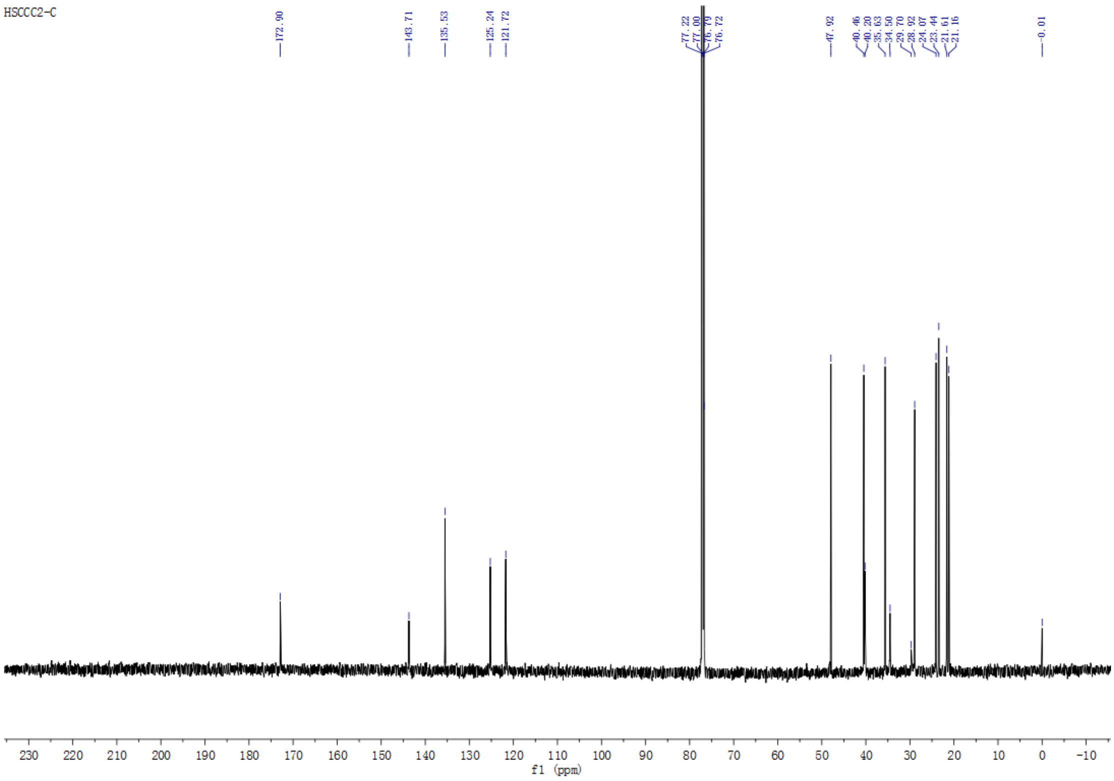

Figure S1. ^{13}C NMR spectrum (600MHz) of compound **1**.

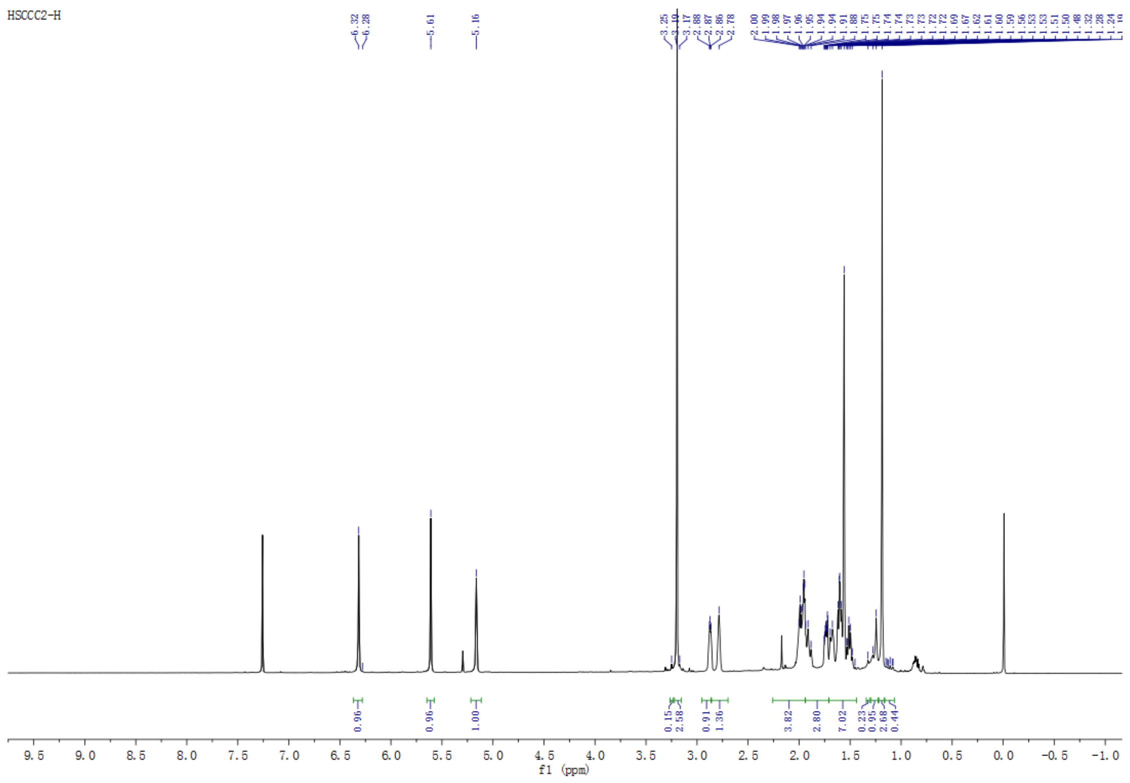

Figure S2. ^{1}H NMR spectrum (600MHz) of compound **1**.

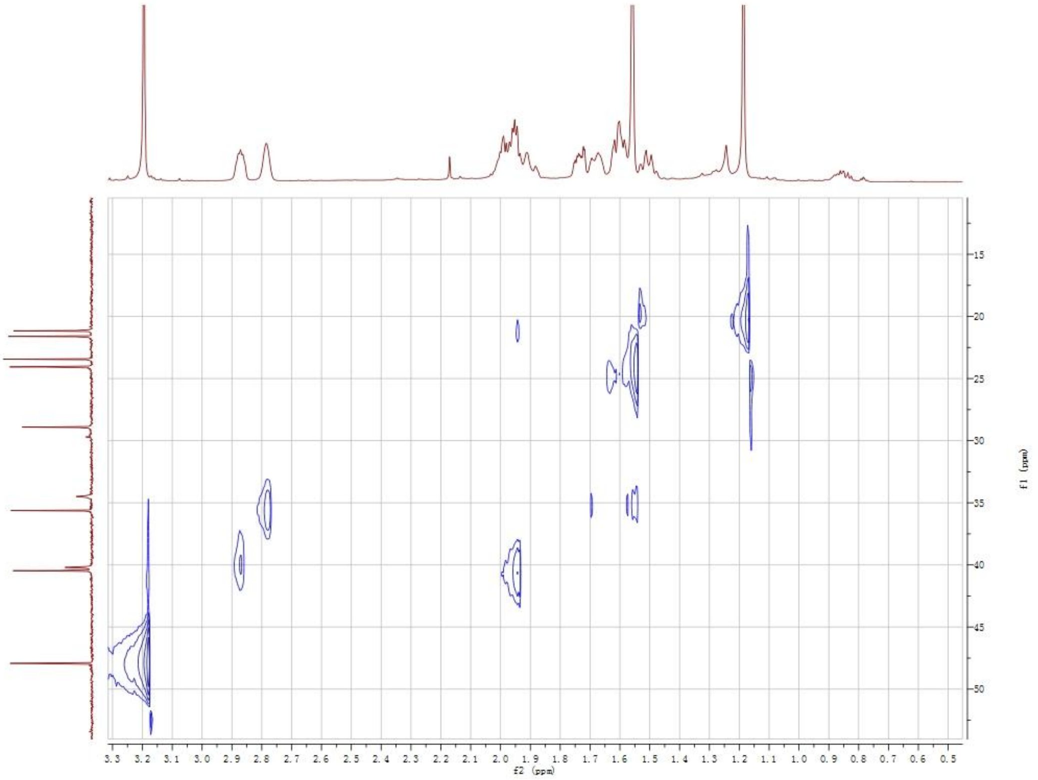

Figure S3. HSQC spectrum (600MHz) of compound **1**.

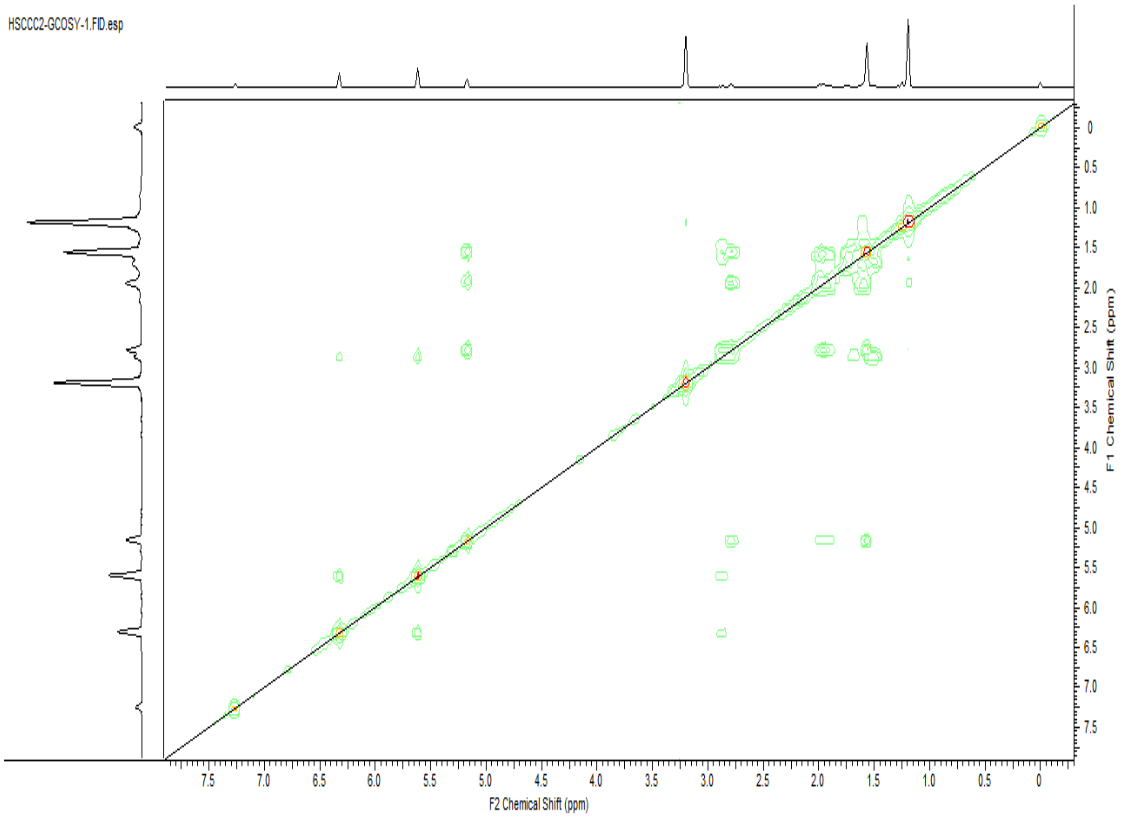

Figure S4. ^1H-^1H COSY spectrum (600MHz) of compound **1**.

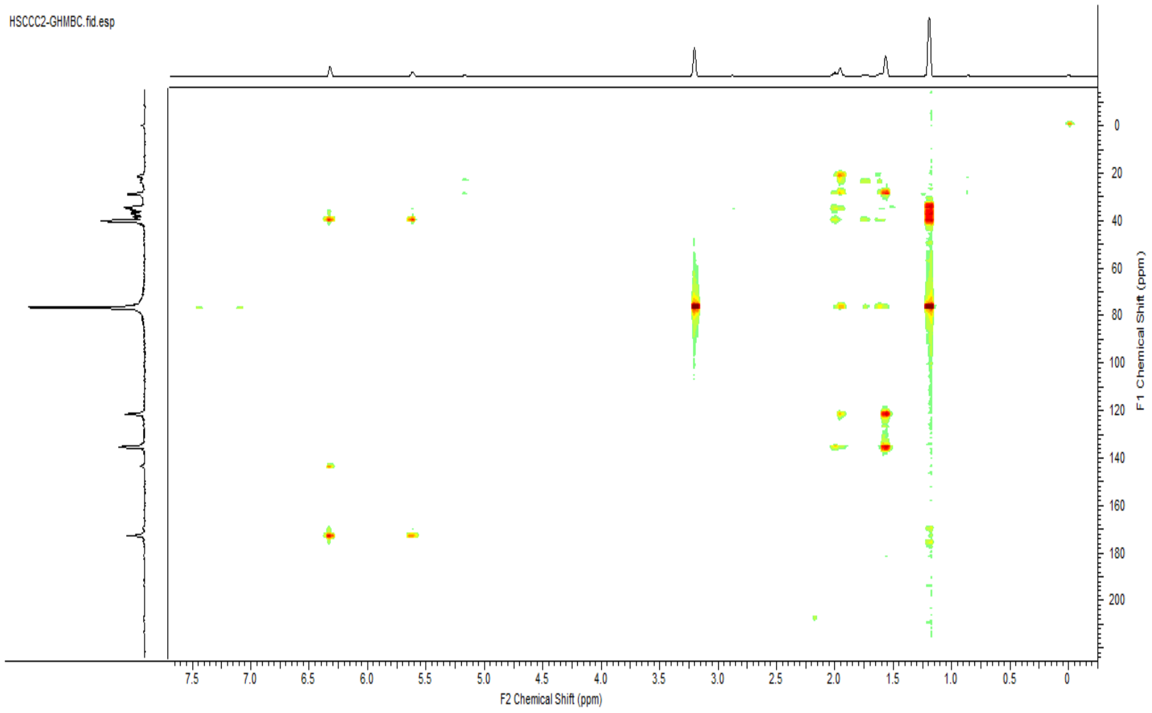

Figure S5. HMBC spectrum (600MHz) of compound **1**.

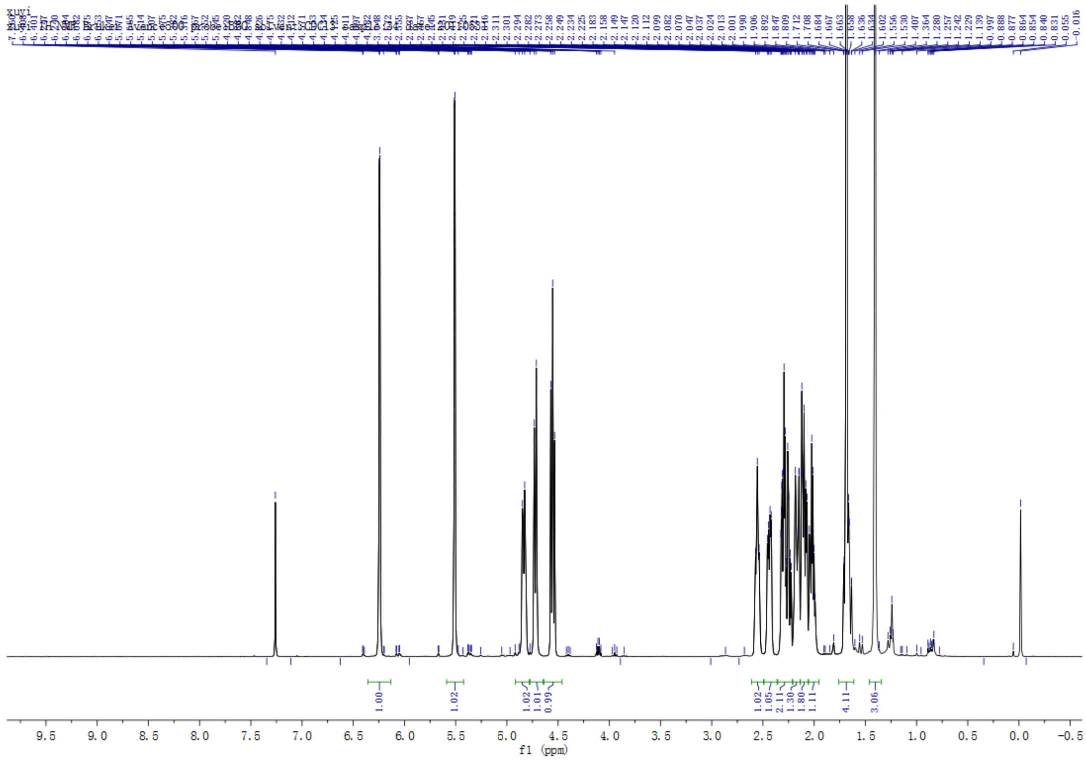

Figure S6. ^{1}H NMR spectrum (500MHz) of compound **2**.

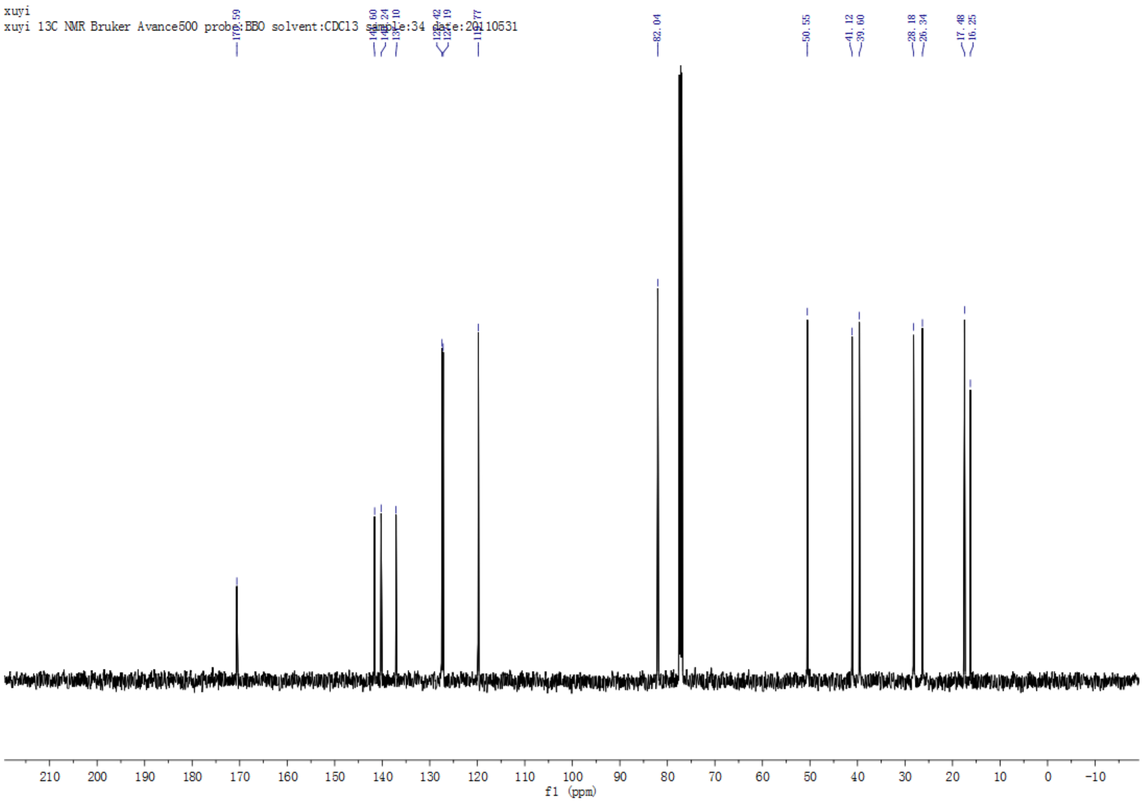

Figure S7. ^{13}C NMR spectrum (500MHz) of compound **2**.

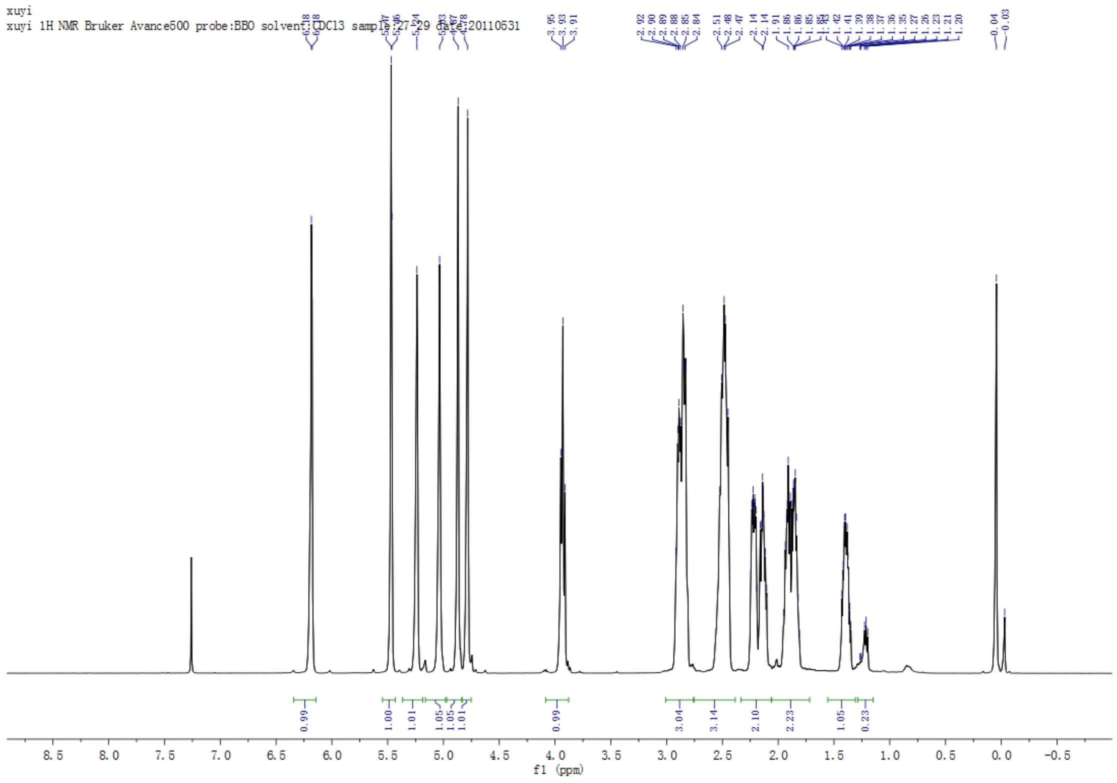

Figure S8. ^{1}H NMR spectrum (500MHz) of compound **3**.

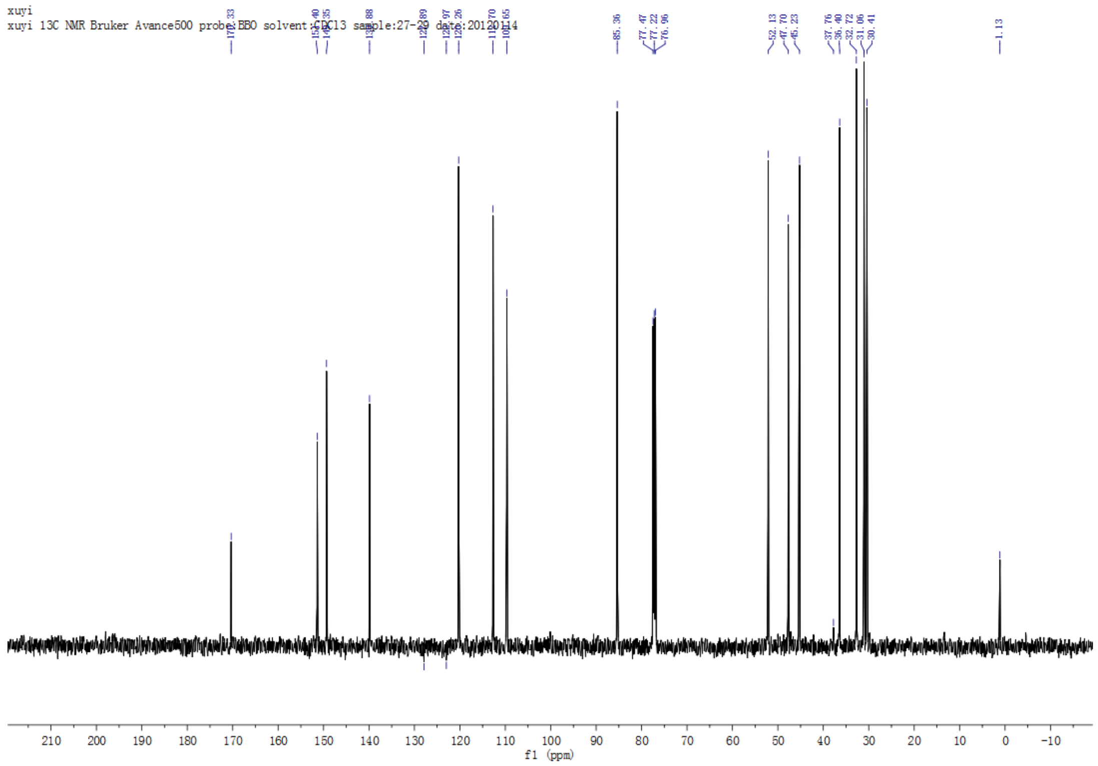

Figure S9. ^{13}C NMR spectrum (500MHz) of compound **3**.

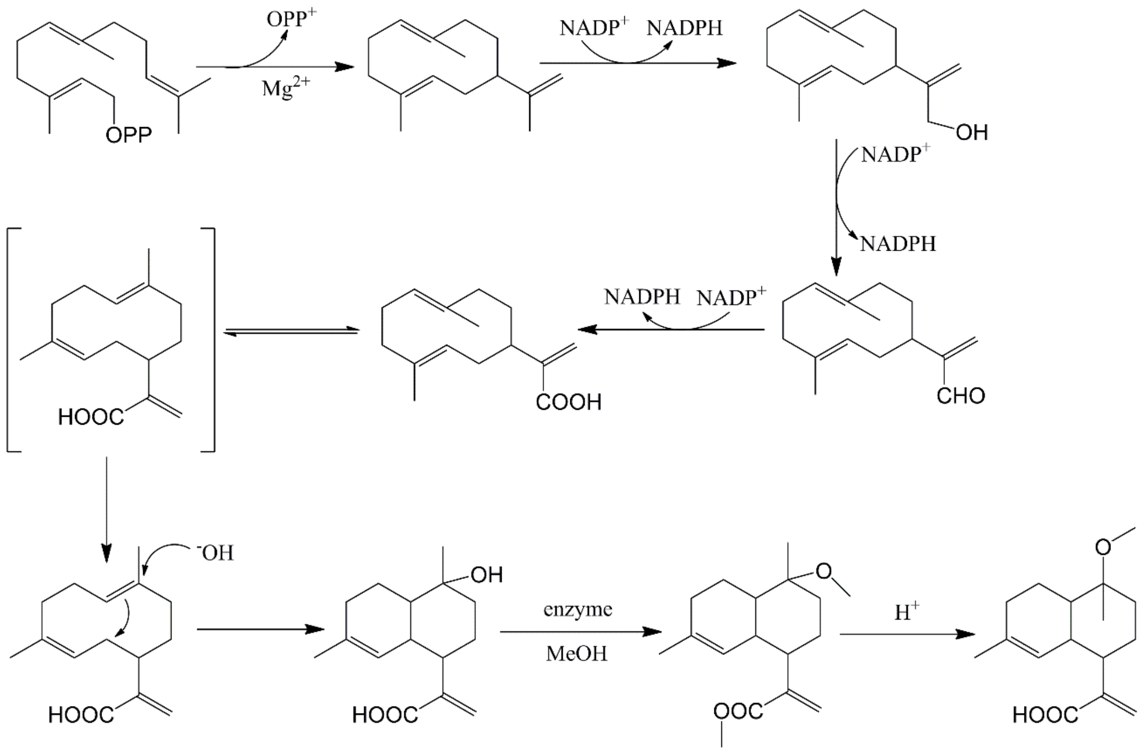

Figure S10. The possible biosynthetic pathway (C) of compound **1**.
